# Desmoplakin Cardiomyopathy: Comprehensive Review of an Increasingly Recognized Entity

**DOI:** 10.3390/jcm12072660

**Published:** 2023-04-03

**Authors:** Mariana Brandão, Riccardo Bariani, Ilaria Rigato, Barbara Bauce

**Affiliations:** 1 Cardiology Department, Centro Hospitalar Vila Nova de Gaia/Espinho, 4430-000 Vila Nova de Gaia, Portugal; 2 Department of Cardiac, Thoracic, Vascular Sciences and Public Health, University of Padova, 35122 Padova, Italy; 3 Azienda Ospedaliera/Universita’ di Padova, Via Giustiniani, 2-Padova, 35128 Padova, Italy

**Keywords:** Desmoplakin, Desmoplakin cardiomyopathy, arrhythmias, sudden cardiac death, arrhythmogenic left ventricular cardiomyopathy

## Abstract

Desmoplakin (DSP) is a desmosomal protein that plays an essential role for cell-to-cell adhesion within the cardiomyocytes. The first association between *DSP* genetic variants and the presence of a myocardial disease referred to patients with Carvajal syndrome. Since then, several reports have linked the *DSP* gene to familial forms of arrhythmogenic (ACM) and dilated cardiomyopathies. Left-dominant ACM is the most common phenotype in individuals carrying *DSP* variants. More recently, a new entity—“Desmoplakin cardiomyopathy”—was described as a distinct form of cardiomyopathy characterized by frequent left ventricular involvement with extensive fibrosis, high arrhythmic risk, and episodes of acute myocardial injury. The purpose of this review was to summarize the available evidence on DSP cardiomyopathy and to identify existing gaps in knowledge that need clarification from upcoming research.

## 1. Background

Desmoplakin (DSP) is a desmosomal protein that is encoded by the *DSP* gene, located on chromosome 6p24. DSP is expressed in both the skin and myocardium and plays an essential role in cell-to-cell adhesion within the cardiomyocytes by anchoring intermediate filaments to the desmosome [1,2].

The first description of a myocardial disease linked to *DSP* mutations referred to patients with Carvajal syndrome, an autosomal recessive cardio-cutaneous syndrome, including dilated cardiomyopathy (DCM), palmoplantar keratoderma and wooly hair [3].

The first report of an association between *DSP* genetic variants and arrhythmogenic cardiomyopathy (ACM) dates back to 2002, when Rampazzo et al. identified a *DSP* missense mutation in a family of Italian ACM patients [4]. Two years later, this link was corroborated by Bauce et al., who reported a series of 38 individuals carrying *DSP* genetic variants, from 4 different ACM families [5]. The authors concluded that familial ACM due to *DSP* variants was characterized by a high occurrence of sudden cardiac death (SCD), and that left ventricular (LV) involvement was not a rare feature. Moreover, chest pain associated with ST segment elevation on basal electrocardiogram (ECG) and myocardial enzyme release, in the setting of angiographically normal coronary arteries, was observed in two cases [5].

In 2007, Sen-Chowdhry and colleagues, in a study including an evaluation of 200 ACM probands by the means of comprehensive cardiac magnetic resonance (CMR) imaging, found a high prevalence of LV involvement (84%), often in the absence of right ventricular (RV) abnormalities [6]. The authors emphasized this peculiar ACM phenotype and coined the term “left-dominant ACM”. Interestingly, in this cohort, carriers of *DSP* variants showed a higher rate of ventricular arrhythmias (VA), which exceeded the degree of systolic disfunction. Notably, myocarditis-like episodes, defined as “hot phases”, were reported [6,7].

Since then, ACM has been characterized by a wide spectrum of RV and LV morphofunctional abnormalities, and three different phenotypes are now recognized: the “classical” right-dominant form (ARVC), with absent or minimal LV involvement; the biventricular phenotype (BIV), with parallel involvement of both ventricles; the left-dominant variant (also defined as arrhythmogenic left ventricular cardiomyopathy, ALVC), without, or only minor, RV abnormalities [6,7,8]. Notably, pathogenic or likely pathogenic (P/LP) variants in the *DSP* gene have been identified in 57% of patients with ALVC [1].

The shifting concept of ACM from an RV or biventricular disease to a possibly isolated LV clinical entity has led to an overlap between ALVC and DCM forms. Indeed, a differential diagnosis between ALVC and DCM is often challenging [9,10], given that they share a genetic background, and since desmosomal gene mutations have also been described in patients with a clinical diagnosis of DCM [11]. However, ACM is mainly characterized by the presence of myocardial fibrosis, which is usually localized in the subepicardial layer of the inferolateral LV segments [12]. In contrast, fibrosis in DCM patients is usually less prominent, and mostly located in the interventricular septum. In order to explain this difference, it has been speculated that in ALVC, fibrosis represents the *primum movens* of the disease, whereas in DCM, it constitutes an epiphenomenon secondary to the dilatation of the ventricle [12,13].

However, the presence of a clinical and pathological overlap between ALVC and DCM can be well exemplified by the fact that, after the first description that considered DCM as the typical cardiac pathology linked to Carvajal syndrome, further detailed examinations of myocardial autoptic specimens of Carvajal patients revealed typical ACM findings—fibrofatty infiltration, aneurysm formation, ECG abnormalities and prominent arrhythmias—with mainly LV involvement [14].

In a genotype–phenotype correlation study in ACM patients with *DSP* mutations, LV structural involvement and dysfunction were significantly more common in non-missense variant carriers [15]. In the same report by Castelletti et al., individuals carrying *DSP* variants showed high arrhythmic risk, and non-missense mutation carriers mostly exhibited left-dominant forms of ACM [15].

Additionally, among patients affected with DCM, *DSP* carriers showed a more malignant and arrhythmogenic phenotype, even in the absence of overt LV dysfunction or dilatation [9,10]. In a multicenter study of 183 genotype positive patients with classic DCM phenotype, desmosomal mutation carriers (mostly *DSP*) experienced higher rates of sudden cardiac death and life-threatening ventricular arrhythmias, even in the presence of a mild depression of the LV ejection fraction (LVEF) [16].

**Figure 1:**
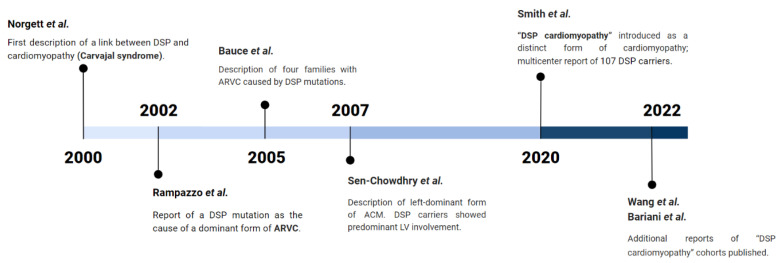
Timeline with landmark studies on DSP cardiomyopathy [3,4,5,6,17,18,19].

Recently, several reports [17,18,19] have described a new entity, named “Desmoplakin cardiomyopathy” (Figure 1), as a distinct form of ACM characterized by peculiar clinical and instrumental features, such as frequent LV involvement with extensive fibrosis, high arrhythmic risk, and episodes of acute myocardial injury.

The purpose of this review is to summarize the available evidence on DSP cardiomyopathy and identify gaps in knowledge that need clarifying by upcoming research.

## 2. Clinical Features

The phenotypic spectrum of DSP cardiomyopathy is wide (Table 1) [20]. The main clinical presentations include extensive subepicardial late gadolinium enhancement (LGE) on CMR, which is often disproportionate to the degree of LV dysfunction, in association with frequent premature ventricular contractions (PVC) and a propensity to life-threatening ventricular arrhythmias [9,17,18,19]. Another cardinal clinical feature of DSP cardiomyopathy is the occurrence of recurrent “myocarditis-like” episodes of chest pain with documented myocardial injury [19,21].

According to the published cohorts, DSP cardiomyopathy is a disease with a female predominance [17,19], an observation contrasting with the male prevalence described in classical forms of ACM. A typical cutaneous phenotype, with wooly hair and/or palmoplantar keratoderma, was reported in a significant percentage of DSP carriers, ranging from 44% to 55% [17,18]. The baseline characteristics of patients from available reports on cohorts of DSP cardiomyopathy are detailed in Table 1.

### 2.1. Performance of ACM Diagnostic Criteria in DSP Cardiomyopathy

As previously mentioned, ALVC is the predominant phenotype in *DSP* carriers. Comprehensibly, the 2010 International Task Force (ITF) Criteria underperformed in patients with DSP cardiomyopathy, since the document was designed for “classical” ACM (with right-dominant or biventricular phenotypes), and ALVC variants were not contemplated [24].

In a study of 38 *DSP* carriers, half of the individuals with cardiac abnormalities failed to comply with diagnostic criteria for ACM [5]. In a multicenter study that compared the clinical features of patients with *DSP* and *PKP2* genetic variants, the 2010 ITF criteria were insensitive for diagnosing ACM in *DSP* carriers (34% vs. 49%, *p* = 0.02), despite the presence of clinically confirmed cardiomyopathy [17].

Wang et al. [19] also found that, in a population of 91 *DSP* carriers, only 51% met the ITF diagnostic criteria at baseline. More importantly, the 2010 ITF criteria failed to identify individuals who presented with arrhythmic and heart failure events during follow-up, showing a limited prognostic value in this cohort [19].

The recognition of the inadequacy of these criteria, which failed to include the entire phenotypic spectrum of the disease [9,24], led to an upgraded consensus document—the 2020 Padua criteria [8]. In this document, the authors addressed the broad range of ACM variants and provided specific criteria for the LV phenotype; additionally, criteria based on CMR tissue characterization findings were included [8,24].

In our *DSP* cohort [18], by applying the 2010 ITF criteria, only 42% of patients reached a definite diagnosis of ACM. However, when using the 2020 Padua criteria [8], 67% of carriers received a definite diagnosis [18], in keeping with a better performance of the Padua criteria in patients with DSP cardiomyopathy.

### 2.2. Myocarditis-like Episodes

The presence of inflammatory cell infiltrates in endomyocardial biopsies or autopsy samples has been reported in ACM patients since the first description of the disease in the early 1980s, thus suggesting a possible role of inflammation in its pathophysiology [5,21].

In individuals with ACM, episodes of chest pain, accompanied by electrocardiographic changes and myocardial enzyme release, in the setting of normal coronary arteries, have been defined as *“hot phases”* [5,15,22]. Bauce et al. were the first to describe the occurrence of clinical myocarditis in two siblings carrying a *DSP* gene variant [5]. It is noteworthy that the occurrence of such episodes, combined with a shared CMR pattern characterized by subepicardial LGE, may lead to difficulties in differential diagnostics with acute and chronic myocarditis.

Recently, Bariani et al. reported a 5% incidence of “*hot phases*” among 560 ACM patient probands followed at our reference center; in 58% of these subjects, CMR showed evidence of myocardial inflammation, according to the Revised Lake Louise criteria [25]. Finally, DSP genetic variants were identified in 39% of cases [25].

The majority of papers reporting the occurrence of *“hot phases”* consists of case reports. In a recent systematic review including 103 ACM patients with these myocarditis-like episodes, *DSP* was also the most frequently implicated gene (69%) [21]. Furthermore, patients were mostly young (mean age 26 ± 14 years) and affected by ALVC forms (68%).

Interestingly, a recent European study was carried out, whereby genetic evaluations were performed on 336 consecutive patients with acute myocarditis—a P/LP variant in ACM- or DCM-associated genes was found in 8% of cases, and desmosomal mutations, mostly *DSP* truncating variants, were found in 3% [26].

A recent multicenter study compared the outcomes of patients with acute myocarditis with and without desmosomal gene variants. In this report, genotype-positive patients (89% DSP variant carriers) had a significantly higher incidence of the main endpoint (death, ventricular arrhythmias, recurrent episodes of myocarditis and heart failure) than genotype-negative individuals (62.3% vs. 17.5% at 5 years, *p* < 0.0001) [27]. Moreover, a family history of myocarditis was significantly more often reported in the desmosomal gene variant group (22.2%) [27].

Across the reports on DSP cardiomyopathy, the prevalence of “hot phases” ranges from 14% to 22% [17,18,19]. Smith et al. found evidence of acute myocardial injury in 14% of DSP cardiomyopathy patients, and four of them had myocardial inflammation documented by 18F-fluorodoxyglucose positron emission tomography [19]. Similarly, in our cohort from Padova, 15% of *DSP* carriers presented episodes of clinically suspected myocarditis during a mean follow-up of 11 years [18]. The highest incidence of myocardial injury was reported by Wang et al. in 22% of DSP cardiomyopathy patients, at a median age of 28 years [19]. In detail, more than 90% of these patients presented LGE on CMR. Interestingly, the authors found myocarditis-like episodes to be associated with HF and ventricular arrhythmic events, and hypothesized that acute myocardial injury episodes could play a role in the development of LV fibrosis and dysfunction in *DSP* carriers [19].

Regardless of their impact on disease progression, myocarditis-like episodes are part of the distinct phenotype of DSP cardiomyopathy, which warrant a careful differential diagnosis with other inflammatory myocardial diseases, such as myocarditis or sarcoidosis [19]. Thus, genetic testing should be considered in individuals presenting with recurrent episodes of acute myocarditis, particularly in the setting of a positive family history for myocarditis or cardiomyopathy.

## 3. Electrocardiographic Features

Electrocardiograms (ECG) of patients with DSP cardiomyopathy mostly reflect the presence of ALVC forms. Typical ECG abnormalities include low QRS voltages (peak to peak < 0.5 mV) in limb leads and T-wave inversion in lateral or inferior leads [13]. These findings indicate the fibrofatty substitution that disrupts the normal myocardium and electrical tissue, mostly in the inferolateral LV regions [5,12]. However, similarly to patients with ALVC, ECG is often unremarkable in DSP cardiomyopathy.

In our *DSP* cohort [18], low QRS voltages in the limb leads was the most common (44%) ECG finding, followed by T-wave inversion in V4–V6 (22%). A high prevalence of negative T waves in precordial leads (46%) in *DSP* carriers was previously reported by our group [5]. Contrary to classical ACM reports, the presence of an epsilon wave was rare (3%) [18].

López-Ayala et al., in a cohort of patients with a founder *DSP* truncating mutation, observed that individuals without signs of LV involvement already presented ECG abnormalities, pointing out that T-wave inversion in inferior leads may be a sensitive marker for ALVC in *DSP* carriers, even in the absence of overt disease [9]. In a multicenter report, T-wave inversion was uncommon in *DSP* probands, but when evident in leads V4–V6, an association with LV involvement was found [17].

**Figure 2:**
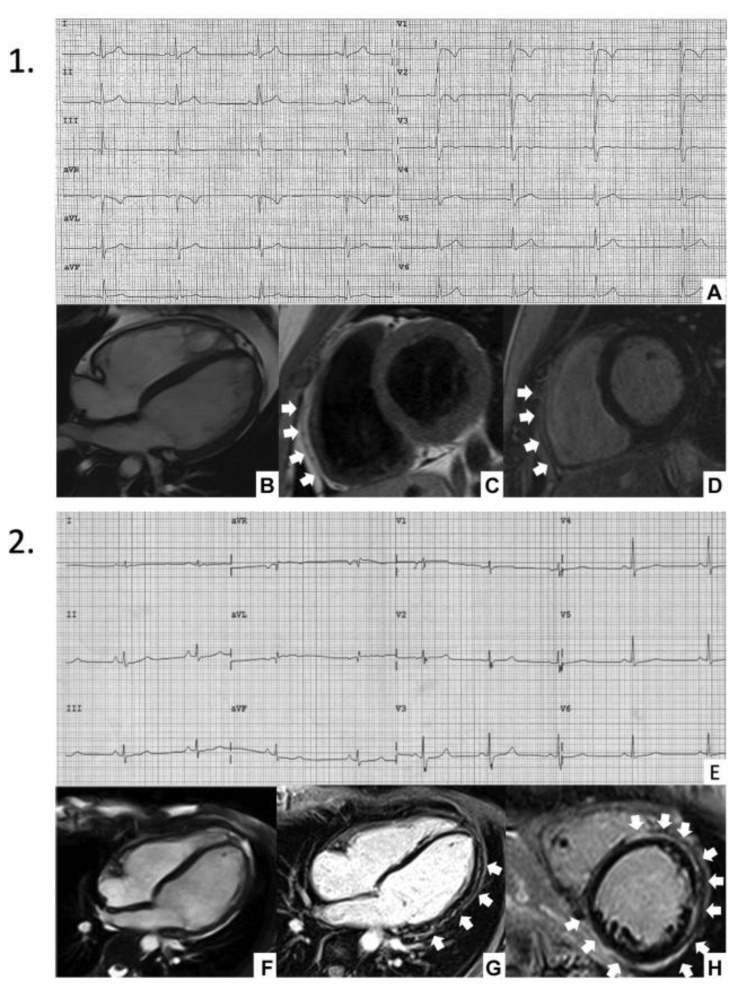
DSP cardiomyopathy. In **Panel 1**, ECG demonstrates the presence of TWI from leads V1–V3 (**A**) and CMR shows mild RV dilatation and moderate dysfunction with anterior and inferior wall akinesia in the mid-apical segments (**B**); in the same regions, fibrofatty infiltration (*white arrows)* was present (**C**,**D**). In **Panel 2**, ECG shows diffuse-low QRS voltages (**E**) and CMR demonstrates a mild LV dilatation with moderate systolic dysfunction (**F**). After gadolinium administration, circumferential LGE *(white arrows)* appeared (**G**,**H**). CMR = cardiac magnetic resonance; ECG = electrocardiography; LGE = late gadolinium enhancement; LV = left ventricle; RV = right ventricle; TWI = T-wave inversion. Adapted from Bariani et al. [18] with permission. The patient consented to the publication of this material.

The presence of low QRS voltages in limb leads and negative T waves in inferolateral leads have been pointed out as ECG markers that could aid in differential diagnosis in patients with overlapping features of ALVC and DCM [12,13] (Figure 2). The ECG features of patients from DSP cardiomyopathy cohorts are summarized in Table 2.

### Ventricular Arrhythmias

Predisposition to VAs is a characteristic feature of DSP cardiomyopathy since its first description [5,9]. One of the earliest reports of *DSP* carriers showed a ventricular arrhythmia rate of 46% [5]. Frequent PVCs (>500 per 24 h) are a prominent clinical feature (≈40–60%) among reports on DSP cardiomyopathy (Table 2). This observation is congruent with the arrhythmogenic phenotype consistently observed in *DSP* carriers.

ALVC-related arrhythmic events comprise PVCs, non-sustained ventricular tachycardia (VT) and sustained VT (usually with right bundle branch block pattern), and ventricular fibrillation (VF) [8,13].

Malignant VAs occurred in 29% of our *DSP* cohort [18] during a mean follow-up of 11 years; among patients with ALVC with life-threatening arrhythmias, all presented normal or mildly reduced LVEF [18], corroborating the disproportionality of the arrhythmic propensity to the degree of LV dysfunction in this subset of patients.

Wang et al. also reported a high rate of malignant VAs (27%), including a substantial portion of patients with VF/aborted sudden death (17%) [19]. The authors found an incidence rate of the arrhythmic endpoint (composite of spontaneous sustained VT, aborted SCD, appropriate implantable cardioverter-defibrillator (ICD) therapy for a sustained VT) of 5.9 per 100 people per year (95% confidence interval [CI]: 3.9–9.1) [19]. A similar severe arrhythmia outcome was previously described in 28% of *DSP* carriers by Smith et al., with a particularly high incidence in individuals with proband status (43%) [17].

## 4. Cardiovascular Imaging Features

Hallmark cardiovascular imaging features in DSP cardiomyopathy are mostly obtained by CMR. ALVC is the most common phenotype in individuals carrying P/LP *DSP* variants [6,18,19]. Considering that in ALVC forms, myocardial fibrosis is usually confined to the epicardial layer, LV dimensions and systolic function are frequently normal. Thus, echocardiographic evaluation can be unremarkable in *DSP* carriers, in spite of a coexisting extensive scar. Wall motion abnormalities and LV dysfunctions detectable by echocardiography may not be present until advanced stages, since the subendocardial layer is usually spared [12].

Therefore, CMR is the most sensitive test for phenotype assessment in *DSP* carriers. In a multicenter study of *DSP* carriers, LGE was detected in a quarter of patients with LV-predominant disease, in the absence of overt dysfunction [17]. A seemingly distinctive feature of DSP cardiomyopathy is the disproportionate involvement of the left ventricle [19] with extensive fibrosis and arrhythmic propensity that largely exceed the degree of ventricular dysfunction [10,17,28].

Among the reviewed DSP cardiomyopathy cohorts, subepicardial LGE in ≥2 LV segments was present in 40% to 72% of patients [17,18,19] (Table 2). Notably, these reports included phenotype-negative family members, which may account for a somewhat large proportion of carriers without tissue characterization abnormalities. Interestingly, López-Ayala et al. found CMR evidence of extensive fibrosis in *DSP* patients, even in young carriers [9].

These findings are in line with a study by Augusto and colleagues, which established an association between a characteristic subepicardial, “ring-like” scar pattern and *DSP*/*FLNC* variants [10], among patients with a clinical label of DCM. This circumferential LGE pattern was found in 84% of *DSP* carriers [10]. In their genotype-imaging phenotype study, the presence of regional wall motion abnormalities, in either the left or right ventricle, was also more common in *DSP*/*FLNC* carriers; the detection of myocardial fat infiltration was exclusive to the *DSP*/*FLNC* phenotypes [10].

They also noted a tendentially higher LVEF in *DSP*/FLNC carriers, in comparison to other DCM genotypes [10]. This is in accordance with our CMR study—ALVC patients presented less depressed LVEF than DCM probands (46% versus 29%, *p* < 0.01) [12].

Apart from the distinctive “ring-like” pattern, the preferential LGE distribution in ALVC, affecting predominantly the inferior wall-septal junction and inferolateral walls, has consistently been described since early reports [6,29], and corresponds to the fibrofatty replacement observed in the subepicardium and midwall of LV specimens [30] (Figure 2).

In DSP cardiomyopathy, LV dysfunction appears to be intimately related to the extent of LV scar [10,12]. Of note, Cipriani et al. showed that 100% of patients with left involvement and reduced LVEF showed LV LGE, with more extensive fibrosis than documented in patients with preserved LV function [12]. Scar is, therefore, *the cause, not the consequence,* of the cardiomyopathic process, and is disproportionate to the level of LV dilation and dysfunction in *DSP* carriers.

## 5. Therapy, Risk Stratification, and Prognosis

Since DSP cardiomyopathy is a recently described entity, extensive data regarding targeted therapy are not available. Current therapy reflects the management of the underlying phenotype (DCM/ALVC), and includes treatment of heart failure, arrhythmias, and the prevention of sudden cardiac death.

DSP cardiomyopathy is accompanied by a high prevalence of VAs, often occurring in young individuals without HF or significant LV dysfunction [9,17,18,19]. However, evidence supporting tailored risk stratification and clinical decision-making in these patients, particularly regarding the primary prevention of SCD, is currently lacking.

LVEF < 55% was strongly associated with malignant VAs in a multicenter *DSP* cohort, regardless of RV involvement [17]. Smith et al. found the classic LVEF < 35% threshold to be an insensitive marker for adverse events in their *DSP* cohort, failing to identify 52% of *DSP* carriers with severe arrhythmias. Indeed, several events occurred in individuals with LVEF of 35–55%, and even with LVEF > 55% [17]. Our study confirmed these findings, revealing also that those presenting with RV involvement (ARVC and BIV forms) showed a higher incidence of arrhythmic events [18]. These observations corroborate the poor discriminative power of LVEF in SCD risk stratification in DSP cardiomyopathy.

It is noteworthy that the 2022 ESC Guidelines for the management of patients with ventricular arrhythmias and the prevention of SCD, while mentioning the increased risk of patients with *LMNA, FLNC, PLN*, and *RBM20* variants in their recommendations, do not include any consideration regarding *DSP* variant carriers [31]. For patients with DCM/hypokinetic non-dilated cardiomyopathy and the before-mentioned genotypes, ICD implantation should be considered for SCD primary prevention, in the presence of LVEF < 50% and another risk factor, such as syncope or LGE on CMR (class of recommendation IIa, level of evidence C) [31]. As demonstrated in genotype–phenotype correlation studies, desmosomal mutation carriers (including *DSP*) had rates of SCD/VT/VF comparable to those of *LMNA* patients [16,32]. In light of the reviewed literature, a higher LVEF threshold should probably also be considered for the adoption of primary prevention strategies in *DSP* patients, as a significant portion of events occur in patients with normal or nearly normal LVEF [17,18,19].

The male gender has been considered an adverse prognostic marker, and was included as a minor risk factor in stratification algorithms in classic ACM [33]. Accordingly, in our cohort, male carriers showed a significantly higher incidence of malignant arrhythmias (52% vs. 24%, *p* = 0.036), HF (31% vs. 3%, *p* = 0.004), and cardiac death (31% vs. 0%, *p* = 0.001) (18). However, the incidence of arrhythmias was not different between genders in two other large studies of DSP cardiomyopathy [17,19].

Wang et al. reported an incidence of HF of 6.6 (95% CI: 4.5–9.8) per 100 people per year among *DSP* carriers; proband status (HR 3.21, 95% CI: 1.37–7.52) and myocardial injury (HR 6.44, 95% CI: 2.61–15.90) were independent predictors of HF in this cohort [19].

In summary, classical variables routinely used for risk estimation in DCM and ACM populations appear inadequate and fail to predict the arrhythmic risk in DSP cardiomyopathy. Dedicated tools to predict risk and guide ICD implantation remain an unmet need in the management of *DSP* carriers.

## 6. Carrier Status and the Role of Exercise in *DSP* Cardiomyopathy

Besides probands, family members carrying *DSP* genetic variants have been considered in published *DSP* cohorts. In the study by Wang et al., which included 51% of patients with normal ventricular function, family members had fewer symptoms, less lateral T-wave inversions, and less LGE on CMR compared to probands [19]. However, and consistently with other reports, PVCs’ burden was similar between probands and family members [17,18,19]. In fact, Smith et al. found a considerably high prevalence of PVCs (43%) and severe VAs (18%) among non-probands [17].

Several studies demonstrated that exercise plays a role in disease progression and increases the risk of malignant arrhythmias and SCD in “classical” ACM forms [34,35,36,37]. Accordingly, ESC Guidelines discourage the participation of individuals with ACM in high-intensity recreational exercise or competitive sports; these recommendations extend to phenotype-negative carriers (class of recommendation III, level of evidence B) [38]. However, patients with LV-predominant disease were underrepresented in most studies supporting these recommendations, which included mostly patients with ARVC. Hence, the effect of sporting activity on the LV-predominant subset has not been extensively examined. Interestingly, in the largest published cohort of DSP cardiomyopathy patients, 53% of ALVC patients were engaged in at least moderate exercise, and no difference in ventricular dysfunction or arrhythmias was found between exercise groups [17]. Currently available evidence is scarce to establish exercise level recommendations in patients with DSP cardiomyopathy.

## 7. Conclusions

Genotype–phenotype correlation studies demonstrated that, among patients carrying DSP pathogenic/likely pathogenic genetic variants, a wide clinical and instrumental spectrum is present. The main clinical findings include extensive subepicardial late gadolinium enhancement on cardiac magnetic resonance, which is often disproportionate to the degree of LV dysfunction, in association with frequent premature ventricular contractions and a propensity to life-threatening ventricular arrhythmias. Furthermore, patients can show recurrent myocarditis-like episodes of chest pain with documented myocardial injury, named “hotphase episodes”.

Evidence supporting tailored risk stratification and clinical decision-making in these patients, particularly regarding primary prevention of sudden cardiac death, is currently lacking.

## Figures and Tables

**Table 1:** Baseline characteristics of patients with DSP cardiomyopathy.

Author/Year	Number of Patients	Age at Diagnosis (Years)	M:F (%)	Index Case	Cutaneous Phenotype ^•^	LV+ Involvement	RV+ Involvement	Normal Ventricular Function	Myocardial Injury *	Frequent PVCs
Smith et al., 2020 [17]	107	36 ± 16 ^†^	31:69	41%	55%	51%	14%	36%	14%	56%
Wang et al., 2022 [19]	91	28 (20–44) ^‡^	33:66	49%	-	28%	6%	51%	22%	48%
Bariani et al., 2022 [18]	73	33 (19–50) ^‡^	47:53	36%	44%	36%	22%	15%	15%	43%
Reza et al., 2022 [22]	19	-	37:63	58%	-	37%	11%	-	-	-
Di Lorenzo et al., 2023 [23]	18	41 (31–47) ^‡^	50:50	100%	-	55%	22%	56%	39%	72%

† Mean ± standard deviation; ‡ Median (25th–75th percentile), • Palmoplantar keratoderma or curly hair, * episodes of chest pain with myocardial enzyme release in the presence of normal coronary arteries; >500/24 h. F: female. LV+: left ventricle predominant. M: male. PVCs: premature ventricular contractions. RV+: right ventricle predominant.

**Table 2:** Electrocardiographic and imaging features of DSP cardiomyopathy.

Instrumental Features	Smith et al., 2020 [17] n = 107	Wang et al., 2022 [19] n = 91	Bariani et al., 2022 [18] n = 73	Reza et al., 2022 [22] n = 19	Di Lorenzo et al., 2023 [23] n = 18
Frequent PVCs	56%	48%	43%	-	72%
TWI V1-V3	8%	20%	21%	16%	-
TWI V4-V6	21%	25%	22%	37%	11%
Low QRS voltage	-	15%	62%	-	33%
LVEF, %	46 ± 14 ^†^	49 ± 13 ^†^	52 (44–63) ^‡^	-	49 (42–56) ^‡^
LV LGE	40%	49%	72%	92%	100%
RVEF, %	-	49 ± 12 ^†^	55 (47–63) ^‡^	-	53 (44–57) ^‡^

† Mean ± standard deviation; ‡ Median (25th–75th percentile). LGE: late gadolinium enhancement. LV: left ventricle. LVEF: left ventricular ejection fraction. PVCs: premature ventricular contractions. RVEF: right ventricular ejection fraction. TWI: T wave inversion.

## Data Availability

The data presented in this study are available on request from the corresponding author.
